# A new pipeline for structural characterization and classification of RNA-Seq microbiome data

**DOI:** 10.1186/s13040-021-00266-7

**Published:** 2021-07-09

**Authors:** Sebastian Racedo, Ivan Portnoy, Jorge I. Vélez, Homero San-Juan-Vergara, Marco Sanjuan, Eduardo Zurek

**Affiliations:** 1 Universidad del Norte, Barranquilla, Colombia; 2 Productivity and Innovation Department, Universidad de la Costa, Calle 58 # 55-56, Barranquilla, Colombia

**Keywords:** Microbial communities, Compositional nature, Classification method, 16 rRNA sequencing

## Abstract

**Background:**

High-throughput sequencing enables the analysis of the composition of numerous biological systems, such as microbial communities. The identification of dependencies within these systems requires the analysis and assimilation of the underlying interaction patterns between all the variables that make up that system. However, this task poses a challenge when considering the compositional nature of the data coming from DNA-sequencing experiments because traditional interaction metrics (e.g., correlation) produce unreliable results when analyzing relative fractions instead of absolute abundances. The compositionality-associated challenges extend to the classification task, as it usually involves the characterization of the interactions between the principal descriptive variables of the datasets. The classification of new samples/patients into binary categories corresponding to dissimilar biological settings or phenotypes (e.g., control and cases) could help researchers in the development of treatments/drugs.

**Results:**

Here, we develop and exemplify a new approach, applicable to compositional data, for the classification of new samples into two groups with different biological settings. We propose a new metric to characterize and quantify the overall correlation structure deviation between these groups and a technique for dimensionality reduction to facilitate graphical representation. We conduct simulation experiments with synthetic data to assess the proposed method’s classification accuracy. Moreover, we illustrate the performance of the proposed approach using Operational Taxonomic Unit (OTU) count tables obtained through 16S rRNA gene sequencing data from two microbiota experiments. Also, compare our method’s performance with that of two state-of-the-art methods.

**Conclusions:**

Simulation experiments show that our method achieves a classification accuracy equal to or greater than 98% when using synthetic data. Finally, our method outperforms the other classification methods with real datasets from gene sequencing experiments.

## Background

Microorganisms living inside and on humans are known as the microbiota. When integrated with their genes’ information, it is known as the microbiome. The Human Microbiome Project (HMP) was an endeavor for the characterization of the human microbiota to further understanding its impact on human health and diseases [1].

In recent years, biological sciences have experienced substantial technological advances that have led to the rediscovery of systems biology [2–4]. These advances were possible thanks to the technological ability to completely sequence the genome from any organism at a low cost [5, 6]. Such advances triggered the development of various analytic approaches and technologies to simultaneously monitoring all the components within cells (e.g., genes and proteins). With the genome information and analytic technologies, the mining and exploration of the resulting data opened up the possibility to better understand biological systems, such as microbial populations, and their complexity. The network structure of such biological systems can give insight into the underlying interactions taking place within those systems [7–10]. Furthermore, the understanding of these interactions can lead to the discovery of new methods that can help physicians, biologists, scientists, and healthcare workers with disease diagnosis, gene identification, classification of new data, and many other tasks [11].

**Fig. 1:**
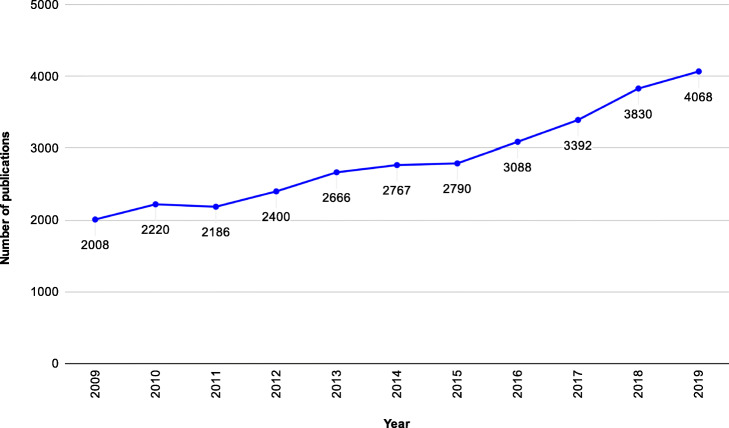
Evolution of the number of publications per year from 2009 to 2019

**Fig. 2:**
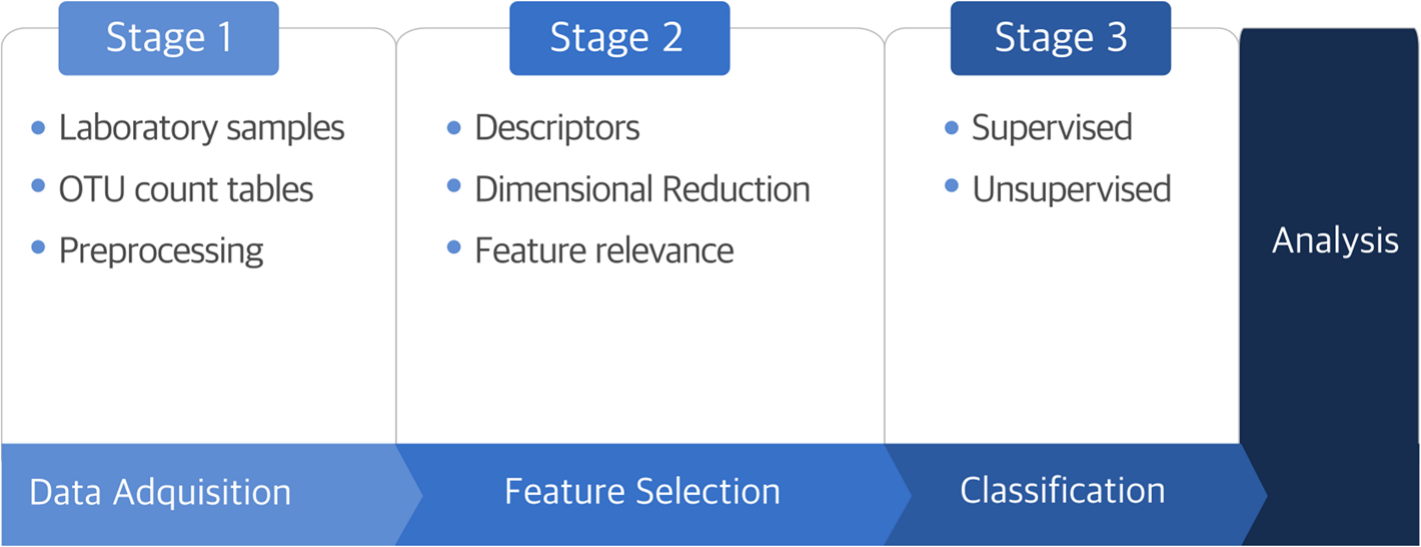
Scheme of gene analysis used for sample classification

We initially conducted a literature search in different medical, biological, and engineering databases as well as academic sites prestigious journals such as BMC Bioinformatics, PLOS ONE, ScienceDirect, and IEEE Xplore using the queries “correlation structure for gene expression classifications,” “classifiers for compositional data,” and “classifiers based on correlation structures” in order to identify papers in English using procedures for sample classification based on correlation structures in the 2009–2019 time window. Figure 1 shows the evolution of the number of publications retrieved when the keywords “correlation structure for gene expression classifications” are used. Publications were retrieved from several academic sites, namely BMC Bioinformatics, PLOS One, ScienceDirect, and Scopus. Figure 2 summarizes the current principal stages of gene expression analysis for sample classification.

Operational Taxonomic Unit (OTU) count tables are the usual output when processing the 16S rRNA sequences of microbiota samples [12]. These tables show the relative abundances of the bacteria that make a microbiota population (e.g., the human gut microbiota). OTU-based data have a compositional nature, which makes them difficult to work with [13, 14]. Thus, data transformation is required prior to any further analysis.

Aitchison [15] proposed two transformations to compensate for the data’s compositionality, thus allowing the use of standard metrics in further analysis. The first transformation is the additive log-ratio (alr), which is defined as:

1 $$ alr\left(\boldsymbol{x}\right)=\left(\mathit{\ln}\frac{x_1}{x_j},\dots, \mathit{\ln}\frac{x_{j-1}}{x_j},\mathit{\ln}\frac{x_{j+1}}{x_j},\mathit{\ln}\frac{x_n}{x_j}\ \right) $$

where *x*_*j*_ is an element of {*x*_1_, *x*_2_, *x*_3_…, *x*_*n*_}. Because one value *x*_*j*_ is selected as the denominator to build the log-ratios, the alr has been criticized as being subjective since the outcome depends mostly on the value of *x*_*j*_ selected [15–18].

The second transformation proposed by Aitchison is the centered log-ratio (clr), which is defined as:

2 $$ clr\left(\boldsymbol{x}\right)=\left[\mathit{\ln}\ \frac{x_1}{g\left(\boldsymbol{x}\right)},\mathit{\ln}\ \frac{x_2}{g\left(\boldsymbol{x}\right)},\dots, \mathit{\ln}\ \frac{x_n}{g\left(\boldsymbol{x}\right)}\ \right] $$

where $g\left(\boldsymbol{x}\right)={\left({\prod}_{i=1}^n{x}_i\right)}^{\frac{1}{n}}$ is the geometric mean. The use of *g*(***x***) avoids the subjectivity of the alr transformation since the method is taking all the information of ***x*** [15–19]. The clr transformation has proven to be reliable and has been extensively used in the scientific literature over the years to analyze microbiome data.

In [20] authors proposed a transformation called the isometric log-ratio (irl) transformation. This approach takes any compositional data ***x*** ∈ *S*^*N*^, and computes *ilr*(***x***) = *z* = [*z*_1_, *z*_2_, …, *z*_*N*_], where *z*_*i*_ is calculated as:

3 $$ {z}_i=\sqrt{\frac{N-i}{N-i+1}\mathit{\ln}\ \left(\frac{x_i}{\sqrt[N-i]{\prod_{j=i+1}^N{x}_j}}\right),}\kern0.75em i=1,..,N. $$

However, implementing the ilr transformation poses serious practical difficulties for high-dimension data as the computational complexity increases rapidly with dimensionality [21].

### Feature selection

After transforming the data, the next step is to separate the data into train, test, and validation sets, although in some cases only the train and test sets are considered. One of the most common problems prior to that step is the limitation of the number of data samples. Indeed, for a normal classifier to be employed using multivariate metrical techniques, the sample size required for optimum training is in order of thousands. This is known as the “curse of dimensionality” problem, and the usual way to overcome this limitation is by using a dimensionality reduction technique to collapse all the attributes (variables) into a lower-dimension space where the most dominant information of the dataset can be retrieved [13, 22].

Feature selection methods are usually separated into three categories: filter, wrapper, and embedded. Table 1 summarizes different approaches for feature selection in gene expression data, the most relevant categories for feature selection, and the current weaknesses when analyzing gene expression data. Filter methods can work with univariate and multivariate data, where univariate methods focus on each feature separately and multivariate methods focus on finding relationships between features [23, 24]. Here we only consider multivariate methods.

**Table 1:** Summary of feature selection approaches in gene expression analysis

Category	Description	Weaknesses	References
*Filter*	- Extract features from the data without any type of learning involved.	- Ignore interaction with the classifier.	[13, 23, 25–30]
*Wrapper*	- Use learning approaches to evaluate which features are useful.	- Risk of overfitting. - Classifier dependent selection.	[23, 26, 29, 30]
*Embedded*	- Combine the traditional feature selection step with the classifier construction.	- Classifier dependent selection.	[23, 26, 29–31]

The abovementioned filter methods tend to be computationally efficient. Wrapper methods, on the other hand, tend to have a better performance in selecting features since they take a model hypothesis into account, meaning that a training and testing procedure is made in the feature space. However, this approach is computationally inefficient and is more problematic as the feature space grows [23, 26, 29, 30]. Embedded methods make the feature selection based on the classifier (i.e., selected features might not work with any other classifier) and hence tend to have a better computational performance than wrappers. This is the case because the optimal set of descriptors is built when the classifier is constructed and the feature selection is affected by the hypotheses made by the classifier [23, 26, 29–31].

In [14], authors presented SParse InversE Covariance Estimation for Ecological ASsociation Inference (SPIEC-EASI), a novel strategy to infer networks from a high dimensional community compositional data. SPIEC-EASI estimates the interaction graph from the transformed data using either Recursive Feature selection or Sparse Inverse Covariance selection and seeks to infer an underlying graphical model using conditional independence. In [32] authors proposed a modification of the Support Vector Machine – Recursive Feature Elimination (SVM-RFE) algorithm for feature selection. SVM-RFE removes one irrelevant feature at each iteration, but this can be troublesome when the number of features is large. Thus, its modification, namely Correlation based Support Vector Machine – Recursive Multiple Feature Elimination (CSVM-RMFE), finds the correlated features and removes more than one irrelevant feature per iteration. Rao and S. Lakshminarayanan [13] presented a new significant attribute selection method based on the Partial Correlation Coefficient Matrix (PCCM).

### Classification

The final step after finding the most relevant features of the transformed data is to select a classifier. In clinical and bioinformatic research, prediction models are extensively used to derive classification rules useful to accurately predict whether a patient has or would develop a disease, whether the treatment is going to work, or even whether a disease would recur [33–35]. Table 2 summarizes the relevant aspects of some widely used classifiers.

**Table 2:** Summary of classifiers used in gene expression analysis

Category	Classifier	References
*Metrical and classical*	- Probabilistic: Bayesian classifier, probabilistic linear discriminant analysis. - Non probabilistic: Support Vector Machine (SVM), SVM-RFE, Nearest-neighbor (NN), linear discriminant analysis.	[13, 37–41]
*Artificial Intelligence*	- Fuzzy Logic, Genetic Algorithms, Classification and Regression trees.	[13, 38, 39, 42, 43]
*Boosting*	- LogitBoost, AdaBoost.M1, GradientBoosting (GrBoost)	[13, 14, 38, 39, 44]

Depending on the data, a classifier can belong to one of two groups: supervised or unsupervised [36]. In supervised classification (learning), samples are labeled according to some a priori-defined classes or categories, whereas in unsupervised learning, samples are not labeled, and the classifier clusters the data into different classes or categories after maximizing or minimizing a set of criteria.

Dembélé and Kastner [37] presented a new Fold Change method that can detect differentially expressed genes in microarray data. The traditional fold change method works by calculating the ratio between the averages from the samples (usually two different biological conditions, e.g., control and case samples). Then, cutoff values (e.g., 0.5 for down- and 2 for up-regulated) are used to select genes under/above such thresholds. This new approach is more accurate and faster than the traditional method and can assign a metric to each differentially expressed gene, which can be used as a selection criterion.

Belciug and F. Gorunescu [43] proposed a novel initialization of a single hidden layer feedforward neural network’s input weights using the knowledge embedded in the connections between variables and class labels. The authors expressed this by the non-parametric Goodman-Kruskal Gamma rank correlation instead of the traditional random initialization. The use of this correlation also helped to increase computational speed by eliminating unnecessary features based on the significance of the rank correlation between variables and class labels.

In [42], authors proposed a framework to find information about genes and to classify gene combinations belonging to its relevant subtype using fuzzy logic, which adapts numerical data (input/output pairs) into human linguistic terms, offering good capabilities to deal with noisy and missing data. However, defining the rules and membership functions might require a lot of prior knowledge from a human expert [41]. Dettling and P. Bühlmann [44] proposed a boosting method combining a dimensionality reduction step with the LogitBoost algorithm [45] and compared it to AdaBoost.M1 [46], the nearest neighbor classifier [47], and classification and regression trees (CART) using gene expression data [48]. Dettling and P. Bühlmann showed that, for low dimensional data, LogitBoost can perform slightly better than AdaBoost.M1, and that for real high dimensional data, their approach can outperform the other classifiers in some cases.

In this paper, we present a new method to classify samples into two groups with different characteristics (i.e., phenotypes, health condition, among others) when data of compositional nature is available. Our method relies on a new metric to quantitatively characterize the overall correlation structure deviation when comparing the two datasets and a new dimensionality reduction approach. The proposed method is assessed and compared, based on classification accuracy, to two state-of-the-art methods using both synthetic datasets and real datasets from RNA-16s sequencing experiments.

## Proposed classification method

Here, we explain in detail the proposed classification method. First, in section “Data pretreatment”, we introduce the Data Pretreatment stage, and in section “Assessing correlation structure distortion”, a novel metric to be used as the metric to assess correlation structure distortion is described. Finally, in section “Dimensionality reduction technique”, we present the proposed classification rule, which is based on the previously defined metric and a proposed dimensionality-reduction approach to assess the disruption of a dataset’s correlation structure after a new sample is included.

### Data pretreatment

Let ${X}_c^{\rho}\in {\mathbb{R}}^{n_c\times m}$ and ${X}_v^{\rho}\in {\mathbb{R}}^{n_v\times m}$ be the OTU count tables where *m* features are assessed in *n*_*c*_ and *n*_*v*_ samples from control and case individuals, respectively. In the expressions above, the superindex *ρ* indicates the datasets are ‘raw’ or without pretreatment. From now on, ${X}_g^{\rho }$ will represent any of the two groups (*g* = *c* for control, or *g* = *v* for case).

When analyzing OTU counts tables, a log-ratio transformation, such as the clr, is to be applied [15, 18, 19] before estimating correlations. However, in order to apply the log-ratio transformation, it is necessary to consider that compositional count datasets may contain null values resulting from insufficiently large or non-existing samples. As log-ratio transformations require data with exclusively positive values, the use of a zero-replacement method is a must. Here we use the Bayesian-multiplicative (BM) algorithm proposed by Martín-Fernández [49]. Let ${\boldsymbol{x}}_{p_i}$ ∈*ℝ*^1 × *m*^  be the *i*-th row of the matrix ${X}_g^{\rho }$ (*i* = 1, 2, …, *n*_*g*_). The BM algorithm replaces the null counts by

4 $$ BM\left({x}_{p_{i,j}}\right)=\left\{\begin{array}{c}{t}_{i,j}\left(\frac{s_i}{n+{s}_i}\right),\kern1em \mathrm{if}\kern0.5em {x}_{p_{i,j}}=0\\ {}{x}_{p_{i,j}}\left(1-\sum \limits_{\forall k\mid {x}_{p_{i,j}}=0}{t}_{i,k}\left(\frac{s_i}{n+{s}_i}\right)\right),\kern0.75em \mathrm{if}\kern0.5em {x}_{p_{i,j}}\ne 0\end{array}\right. $$

When using the Bayes-Laplace prior, we set $n=\sum \limits_{j=1}^m{x}_{p_{i,j}}$, *t*_*i*, *j*_ = *m*^−1^ and *s*_*i*_ = *m*.  Let ${X}_g^{BM}:= BM\left({X}_g^{\rho}\right)$ be the resulting matrix after the BM algorithm is applied row-wise to ${X}_g^{\rho }$.

To ensure the data’s compositionality on ${X}_g^{BM}$, a closure operation [15, 18, 19] is applied to every row of ${X}_g^{BM}$, as follows:

5 $$ c\left({\boldsymbol{x}}_{p_i}^{BM}\right)=\frac{k}{\sum \limits_{j=1}^m{x}_{p_{i,j}}^{BM}}{\boldsymbol{x}}_{p_i}^{BM} $$

where *k* is an arbitrary constant (usually *k* = 100). Let ${X}_g^{BM,c}:= c\left( BM\left({X}_g^{\rho}\right)\right)$ be the resulting matrix after the BM algorithm and the closure operation have been applied. Now, the clr transformation is applied to each vector ***x***_*p*_ ∈ *ℝ*^1 × *n*^ ${X}_g^{BM,c}$, as

6 $$ clr\left({\boldsymbol{x}}_p\right)=\left[\ln \frac{x_1}{g\left({\boldsymbol{x}}_p\right)},\ln \frac{x_2}{g\left({\boldsymbol{x}}_p\right)},\dots, \ln \frac{x_n}{g\left({\boldsymbol{x}}_p\right)}\right] $$

where $g\left({x}_p\right)={\left({\prod}_{i=1}^n{x}_i\right)}^{\frac{1}{n}}$ is the geometric mean. Hence,

7 $$ {X}_g= clr\left(c\left( BM\left({X}_g^{\rho}\right)\right)\right) $$

Finally, a normalization is applied, resulting in:

8 $$ {X}_{g_{norm}}=\left({X}_g-{I}_{n_g}{b}_g^T\right){\varSigma}_g^{-1} $$

where ${I}_g=\left[1\ 1\dots .1\right]\in {\mathbb{R}}^{n_g\times 1}$ is a column vector of ones, ${b}_g\in {\mathbb{R}}^{n_g\times 1}$ is a column vector that contains the means of all the variables in *X*_*g*_, and *Σ*_*g*_ ∈ *ℝ*^*m* × *m*^ is a diagonal matrix that contains the standard deviation (${\sigma}_{g_i}$, for *i* = 1, …, m) of all variables.

### Assessing correlation structure distortion

Here, we introduce *φ*, a new metric to quantitatively assess the distortion in the correlation structure of a dataset after the incorporation of a new sample. The Pearson correlation matrix for *X*_*g*_ is calculated as follows [50]:

9 $$ {S}_g=\frac{1}{n_g-1}\ {X}_{g_{norm}}^T\ {X}_{g_{norm}} $$

Now, consider a new sample, ***x***_*p*_ ∈ *ℝ*^1 × *m*^. The pretreatment step for this sample yields:

10 $$ {\boldsymbol{x}}_p= clr\left(c\left( BM\left({\boldsymbol{x}}_p\right)\right)\right) $$

**Fig. 3:**
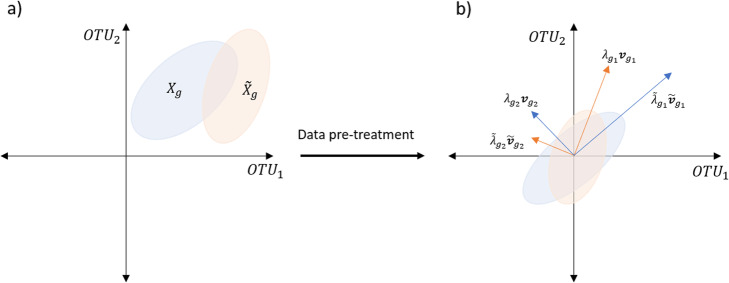
Bidimensional representation of datasets ${\overset{\sim }{X}}_g$ and *X*_*g*_ **a** without pretreatment, and **b** after the pretreatment along with the eigenvectors scaled by the corresponding eigenvalues

Let ${\overset{\sim }{X}}_g{\mathbb{R}}^{n_g\times m}$ be the (augmented) dataset *X*_*g*_ after incorporating the new sample, and let *S*_*g*_ and ${\overset{\sim }{S}}_g$ be the correlation matrices for *X*_*g*_ and ${\overset{\sim }{X}}_g$, respectively. The spectral decomposition for these matrices is

11 $$ {S}_g={V}_g{\Lambda}_g{V}_g^T,\kern3.75em {\overset{\sim }{S}}_g={\overset{\sim }{V}}_g{\overset{\sim }{\Lambda}}_g{\overset{\sim }{V}}_g^T $$

where

12 $$ {\Lambda}_g=\left[\begin{array}{ccc}{\lambda}_{g_1}& & \\ {}& \ddots & \\ {}& & {\lambda}_{g_m}\end{array}\right]\in {\mathbb{R}}^{m\times m},\kern0.5em {\overset{\sim }{\Lambda}}_g=\left[\begin{array}{ccc}{\overset{\sim }{\lambda}}_{g_1}& & \\ {}& \ddots & \\ {}& & {\overset{\sim }{\lambda}}_{g_m}\end{array}\right]\in {\mathbb{R}}^{m\times m} $$

are diagonal matrices containing the eigenvalues for *S*_*g*_ and ${\overset{\sim }{S}}_g$. Let $$ {V}_g=\left[{\boldsymbol{v}}_{g_1}\kern0.5em {\boldsymbol{v}}_{g_2}\kern0.5em \begin{array}{cc}\cdots & {\boldsymbol{v}}_{g_m}\end{array}\right]\in {\mathbb{R}}^{m\times m} $$ and $$ {\overset{\sim }{V}}_g=\left[\begin{array}{cc}{\overset{\sim }{\boldsymbol{v}}}_{g_1}& {\overset{\sim }{\boldsymbol{v}}}_{g_2}\end{array}\kern0.5em \begin{array}{cc}\cdots & {\overset{\sim }{\boldsymbol{v}}}_{g_m}\end{array}\right]\in {\mathbb{R}}^{m\times m} $$ be the eigenvector matrices of *S*_*g*_ and ${\overset{\sim }{S}}_g$. Figure 3a illustrates, in a 2-dimensional example, the datasets *X*_*g*_ and ${\overset{\sim }{X}}_g$. Figure 3b illustrates the datasets after carrying out the pre-treatment, along with their eigenvectors (which are unitary) scaled by their corresponding eigenvalues obtained from the spectral decompositions. Note that scaled eigenvectors mark out the directions of largest variability, capturing high order interactions between the OTUs ruling the overall association structure. Therefore, looking at deviations in both the magnitude and direction of those scaled eigenvectors must give insightful information on overall changes in the association structure of a microbiota population.

Based on the abovementioned remarks, we introduce *φ* to characterize the distortion produced in the underlying correlation structure when two OTU counts datasets are compared. This metric first requires a dimensional reduction, which will be performed by selecting the principal components for each sample group. This procedure, integrated within the Principal Component Analysis (PCA) algorithm [25], consists of finding the minimum number of eigenvalues *a*_*g*_ or $\tilde{a}_{g}$ (for *X*_*g*_ and ${\overset{\sim }{X}}_g$, respectively) that explain 100(1 − *α*)% of the total variance, i.e.:

13 $$ \frac{\sum \limits_{i=1}^{a_g}{\lambda}_{g_i}}{\sum \limits_{i=1}^m{\lambda}_{g_i}}\le \left(1-\alpha \right),\kern3.75em \frac{\sum \limits_{i=1}^{\tilde{a}_{g}}{\lambda}_{g_i}}{\sum \limits_{i=1}^m{\lambda}_{g_i}}\le \left(1-\alpha \right) $$

Thus, *φ* is defined as

14 $$ \varphi =\sum \limits_{j=1}^{\max \left({a}_g,\tilde{a}_{g}\right)}\left[\max \left\{{\lambda}_{g_j},{\overset{\sim }{\lambda}}_{g_j}\right\}\ \left({\lambda}_{g_j}-{\overset{\sim }{\lambda}}_{g_j}\right)\ {\cos}^{-1}\left({\boldsymbol{v}}_{g_j}^T{\overset{\sim }{\boldsymbol{v}}}_{g_j}\right)\right] $$

where $\left({\lambda}_{g_j}-{\overset{\sim }{\lambda}}_{g_j}\right)$ is the algebraic difference (magnitude deviation) of the *j*-th eigenvalues in Λ_*g*_ and ${\overset{\sim }{\Lambda}}_g$, ${\cos}^{-1}\left({\boldsymbol{v}}_{g_j}^T{\overset{\sim }{\boldsymbol{v}}}_{g_j}\right)$ computes angular deviation between the *j*-th eigenvectors in V_*g*_ and ${\overset{\sim }{V}}_g$, and $\max \left\{{\lambda}_{g_j},{\overset{\sim }{\lambda}}_{g_j}\right\}$ provides a weighting factor so that the contribution of the *j*-th deviation to the index *φ* is proportional to the relative importance among principal components.

### Dimensionality reduction technique

Now that we have a metric to measure the distortion caused in the correlation structure of the *g* group after the incorporation of a new sample, we could then infer to which group the new sample would belong, providing a classification criterion based on how distorted the correlation structure is when incorporating ***x***_*p*_. The intuitive way of approaching the evaluation of the distortion would be to integrate ***x***_*p*_ into *X*_*g*_ and (re)calculate the correlation matrix for the further evaluation of its distortion. However, considering that the *g* group may contain many samples, a single new sample may not be enough to generate a significant distortion in the correlation structure. Furthermore, if the number of samples in the groups is unbalanced, the distortion caused by the inclusion of a new sample may not be comparable.

An approach to overcome this dimensional problem is to randomly subsample a small number of rows in *X*_*g*_, combining them with ***x***_*p*_, and then calculating the distortion caused. This approach, however, would not include a considerable amount of information, which is contained in the rows that were left out. To address this issue, we propose a new dimensionality reduction approach that allows a weighted assessment of the distortion in *S*_*g*_ caused by the integration of a new sample ***x***_*p*_. This approach will use all the information contained in the original data, with the objective of providing a classification algorithm for any upcoming sample.

The first step of the proposed approach is to find an expression for the distorted correlation matrix that reveals the natural weights of the contributions of *X*_*g*_ and ***x***_*p*_ to the make-up of the new correlation structure. Suppose that the data is concatenated as:

15 $$ {\overset{\sim }{X}}_g=\left[\begin{array}{c}{X}_g\\ {}{\boldsymbol{x}}_p\end{array}\right]{\mathbb{R}}^{\tilde{n}_{g}\times m} $$

where $\tilde{n}_{g}={n}_g+1$ is the number of rows of ${\overset{\sim }{X}}_g$. Combining Eqs. (15) and (8) yields

16 $$ {\overset{\sim }{X}}_g=\left[\begin{array}{c}{X}_{g_{norm}}{\Sigma}_g+{I}_{n_g}{b}_g^T\\ {}{\boldsymbol{x}}_p\end{array}\right] $$

Normalizing $$ {\overset{\sim }{X}}_g $$ produces

17 $$ {\overset{\sim }{X}}_{g_{norm}}=\left({\overset{\sim }{X}}_g-{I}_{\tilde{n}_{g}}\tilde{b}_{g}^T\right){\overset{\sim }{\Sigma}}_g^{-1}=\left[\begin{array}{c}\left({X}_{g_{norm}}{\Sigma}_g-{I}_{n_g}{\Delta b}_g^T\right){\overset{\sim }{\Sigma}}_g^{-1}\\ {}{\boldsymbol{x}}_{p_{norm}}\end{array}\right] $$

where $\tilde{b}_{g}$ is the vector that contains the means of ${\overset{\sim }{X}}_g$, ${\overset{\sim }{\Sigma}}_g$ is a diagonal matrix that contains the distorted standard deviations, $\Delta  {b}_g:= \tilde{b}_{g}-{b}_g$ is the distortion in the mean vector, and ${\boldsymbol{x}}_{p_{norm}}=\left({\boldsymbol{x}}_p-\tilde{b}_{g}^T\right){\overset{\sim }{\Sigma}}_g^{-1}$. Both $\tilde{b}_{g}$ and ${\overset{\sim }{\Sigma}}_g$ are unknown. Thus, we need to derive expressions for them. The distorted means vector is calculated as $\tilde{b}_{g}=\frac{1}{\tilde{n}_{g}}{{\overset{\sim }{X}}_g}^T{I}_{\tilde{n}_{g}}$, which can be converted into:

18 $$ \tilde{b}_{g}=\frac{n_g}{n_g+1}{b}_g+\frac{1}{n_g+1}{\boldsymbol{x}}_p^T $$

Equation (18) shows that the natural weights are ${w}_1=\frac{n_g}{n_g+1}$ and ${w}_2=\frac{1}{n_g+1}$ for *b*_*g*_ and ***x***_*p*_, respectively. To find an expression for the diagonal matrix of distorted standard deviations, ${\overset{\sim }{\Sigma}}_g$, a column-wise subtraction of the mean vector for ${\overset{\sim }{X}}_g$ is performed:

19 $$ {\overset{\sim }{X}}_{g_{mean- centered}}= {\overset{\sim}{X}}_g-{I}_{\tilde{n}_{g}}\tilde{b}_{g}^T=\left[\begin{array}{c}{X}_g-{I}_{n_g}\tilde{b}_{g}^T\\ {}{\boldsymbol{x}}_p-\tilde{b}_{g}^T\end{array}\right] $$

Adding and subtracting ${I}_{n_g}{b}_g^T$ to ${X}_g-{I}_{n_g}\tilde{b}_{g}^T$ in Eq. (19) yields:

20 $$ {\overset{\sim }{X}}_{g_{mean- centered}}=\left[\begin{array}{c}\left({X}_g-{I}_{n_g}{b}_g^T\right)-{I}_{n_g}{\Delta b}_g^T\\ {}{\boldsymbol{x}}_p-\tilde{b}_{g}^T\end{array}\right] $$

where

21 $$ {\overset{\sim }{X}}_{g_{mean- centered}}\left(:,i\right)=\left[\begin{array}{c}\left({X}_g\left(:,i\right)-{b}_g(i){I}_{n_g}\right)-\Delta {b}_g(i){I}_{n_g}\\ {}{\boldsymbol{x}}_p(i)-\tilde{b}_{g}(i)\end{array}\right] $$

is the *i*-th column of ${\overset{\sim }{X}}_{g_{mean- centered}}\left(:,i\right)$, the corresponding *i*-th variable. Then, the variance of this *i*-th variable will be ${\overset{\sim }{\sigma}}_{g_i}^2=\frac{1}{\tilde{n}_{g}-1}{\left({\overset{\sim }{X}}_{g_{mean- centered}}\left(:,i\right)\right)}^T\ {\overset{\sim }{X}}_{g_{mean- centered}}\left(:,i\right)$, which can be written as:

22 $$ \left(\tilde{n}_{g}-1\right){\overset{\sim }{\sigma}}_{g_i}^2=\left[\left({X}_g^T\left(:,i\right)-{b}_g(i){I}_{n_g}^T\right)-\Delta {b}_g(i){I}_{n_g}^T\kern0.5em {\boldsymbol{x}}_p(i)-\tilde{b}_{g}(i)\right]\left[\begin{array}{c}\left({X}_g\left(:,i\right)-{b}_g(i){I}_{n_g}\right)-\Delta {b}_g(i){I}_{n_g}\\ {}{\boldsymbol{x}}_p(i)-\tilde{b}_{g}(i)\end{array}\right] $$

Equation (22) can be further expanded as:

23 $$ \left(\tilde{n}_{g}-1\right){\overset{\sim }{\sigma}}_{g_i}^2=\left({X}_g^T\left(:,i\right)-{b}_g(i){I}_{n_g}^T\right)\left({X}_g\left(:,i\right)-{b}_g(i){I}_{n_g}\right)-\left({X}_g^T\left(:,i\right)-{b}_g(i){I}_{n_g}^T\right)\Delta {b}_g(i){I}_{n_g}-\Delta {b}_g(i){I}_{n_g}^T\left({X}_g\left(:,i\right)-{b}_g(i){I}_{n_g}\right)+{\Delta b}_c^2(i){I}_{n_g}^T{I}_{n_g}+{\left({\boldsymbol{x}}_p(i)-\tilde{b}_{g}(i)\right)}^2 $$

Notice that, in this expression, the terms $\left({X}_g^T\left(:,i\right)-{b}_g(i){I}_{n_g}^T\right)\left({X}_g\left(:,i\right)-{b}_g(i){I}_{n_g}\right)=\left({n}_g-1\right){\sigma}_{g_i}^2$, ${I}_{n_g}^T{I}_{n_g}={n}_g$, and $\left({X}_g^T\left(:,i\right)-{b}_g(i){I}_{n_g}^T\right)\Delta {b}_g(i){I}_{n_g}=\Delta {b}_g(i){I}_{n_g}^T\left({X}_g\left(:,i\right)-{b}_g(i){I}_{n_g}\right)$. Then, Eq. (23) can be reduced to:

24 $$ \left(\tilde{n}_{g}-1\right){\overset{\sim }{\sigma}}_{g_i}^2=\left({n}_g-1\right){\sigma}_{g_i}^2-2\Delta {b}_g(i){I}_{n_g}^T\left({X}_g\left(:,i\right)-{b}_g(i){I}_{n_g}\right)+{n}_g{\Delta b}_g^2(i)+{\left({\boldsymbol{x}}_p(i)-\tilde{b}_{g}(i)\right)}^2 $$

Considering that $\tilde{n}_{g}={n}_g+1$ and ${I}_{n_g}^T{X}_g\left(:,i\right)={I}_{n_g}^T\left({b}_g(i){I}_{n_g}\right)={n}_g{b}_g(i)$, it follows that

25 $$ {\overset{\sim }{\sigma}}_{g_i}=\sqrt{\frac{n_g-1}{n_g}{\sigma}_{g_i}^2+{\Delta b}_g^2(i)+\frac{1}{n_g}{\left({\boldsymbol{x}}_p(i)-\tilde{b}_{g}(i)\right)}^2} $$

From Eq. (25), notice that the (distorted) variances of the variables of the group ${\overset{\sim }{X}}_g$ depend on: (1) the original variances in *X*_*g*_, with natural weight $\frac{n_g-1}{n_g}$; (2) the quadratic (mean centered) values of the new sample, ${\left({\boldsymbol{x}}_p(i)-\tilde{b}_{g}(i)\right)}^2$, with natural weight $\frac{1}{n_g}$; and the quadratic values of the distortion in the mean vector, ${\Delta b}_g^2(i)$. Based on equation [25], the standard deviation matrix for all *m* variables is

26 $$ {\overset{\sim }{\Sigma}}_g=\left[\begin{array}{ccc}{\overset{\sim }{\sigma}}_{g_1}& & \\ {}& \ddots & \\ {}& & {\overset{\sim }{\sigma}}_{g_m}\end{array}\right] $$

Having expressions for $\tilde{b}_{g}$ and ${\overset{\sim }{\Sigma}}_g$, it follows that the distorted correlation matrix is calculated as ${\overset{\sim }{S}}_g=\frac{1}{\tilde{n}_{g}-1}{\overset{\sim }{X}}_{g_{norm}}^T{\overset{\sim }{X}}_{g_{norm}}$ . Combining ${\overset{\sim }{S}}_g$ with Eq. (17) yields

27 $$ \left(\tilde{n}_{g}-1\right){\overset{\sim }{S}}_g=\left[{\overset{\sim }{\Sigma}}_g^{-1}\left({\Sigma}_g{X}_{g_{norm}}^T-{\Delta b}_g{I}_{n_g}^T\right)\kern0.5em {\boldsymbol{x}}_{p_{norm}}^T\right]\left[\begin{array}{c}\left({X}_{g_{norm}}{\Sigma}_g-{I}_{n_g}{\Delta b}_g^T\right){\overset{\sim }{\Sigma}}_g^{-1}\\ {}{\boldsymbol{x}}_{p_{norm}}\end{array}\right] $$

It follows that,

28 $$ \left(\tilde{n}_{g}-1\right){\overset{\sim }{S}}_g={\overset{\sim }{\Sigma}}_g^{-1}{\Sigma}_g{X}_{g_{norm}}^T{X}_{g_{norm}}{\Sigma}_g{\overset{\sim }{\Sigma}}_g^{-1}-{\overset{\sim }{\Sigma}}_g^{-1}{\Sigma}_g{X}_{g_{norm}}^T{I}_{n_g}{\Delta b}_g^T{\overset{\sim }{\Sigma}}_g^{-1}-{\overset{\sim }{\Sigma}}_g^{-1}{\Delta b}_g{I}_{n_g}^T{X}_{g_{norm}}{\Sigma}_g{\overset{\sim }{\Sigma}}_g^{-1}+{\overset{\sim }{\Sigma}}_g^{-1}{\Delta b}_g{I}_{n_g}^T{I}_{n_g}{\Delta b}_g^T{\overset{\sim }{\Sigma}}_g^{-1}+{\boldsymbol{x}}_{p_{norm}}^T{\boldsymbol{x}}_{p_{norm}} $$

As ${X}_{g_{norm}}^T{X}_{g_{norm}}=\left({n}_g-1\right){S}_g$, ${\Sigma}_g{X}_{g_{norm}}^T={X}_g^T-{b}_g{I}_{n_g}^T$, ${X}_{g_{norm}}{\Sigma}_g={X}_g-{I}_{n_g}{b}_g^T$, this expression can be expressed as:

29 $$ \left(\tilde{n}_{g}-1\right){\overset{\sim }{S}}_g=\left({n}_g-1\right){\overset{\sim }{\Sigma}}_g^{-1}{\Sigma}_g{S}_g{\Sigma}_g{\overset{\sim }{\Sigma}}_g^{-1}-{\overset{\sim }{\Sigma}}_g^{-1}\left({X}_g^T-{b}_g{I}_{n_g}^T\right){I}_{n_g}{\Delta b}_g^T{\overset{\sim }{\Sigma}}_g^{-1}-{\overset{\sim }{\Sigma}}_g^{-1}{\Delta b}_g{I}_{n_g}^T\left({X}_g-{I}_{n_g}{b}_g^T\right){\overset{\sim }{\Sigma}}_g^{-1}+{n}_g{\overset{\sim }{\Sigma}}_g^{-1}{\Delta b}_g{\Delta b}_g^T{\overset{\sim }{\Sigma}}_g^{-1}+{\boldsymbol{x}}_{p_{norm}}^T{\boldsymbol{x}}_{p_{norm}} $$

Now, as ${X}_g^T{I}_{n_g}={b}_g{I}_{n_g}^T{I}_{n_g}={I}_{n_g}^T{X}_g={I}_{n_g}^T{I}_{n_g}{b}_g^T={n}_g{b}_g$, the second and third terms of Eq. (29) disappear. Then, the distorted correlation matrix ${\overset{\sim }{S}}_g$ is given by

30 $$ {\overset{\sim }{S}}_g=\frac{n_g-1}{n_g}{\overset{\sim }{\Sigma}}_g^{-1}{\Sigma}_g{S}_g{\Sigma}_g{\overset{\sim }{\Sigma}}_g^{-1}+{\overset{\sim }{\Sigma}}_g^{-1}{\Delta b}_g{\Delta b}_g^T{\overset{\sim }{\Sigma}}_g^{-1}+\frac{1}{n_g}{\boldsymbol{x}}_{p_{norm}}^T{\boldsymbol{x}}_{p_{norm}} $$

Note that, in this expression, ${\overset{\sim }{S}}_g$ depends on three terms:

1. ${\overset{\sim }{\Sigma}}_g^{-1}{\Sigma}_g{S}_g{\Sigma}_g{\overset{\sim }{\Sigma}}_g^{-1}$, which considers the contributions made from the non-distorted correlation matrix *S*_*g*_ after an actualization of the standard deviation, with a natural weight of $\frac{n_g-1}{n_g}$.
2. ${\boldsymbol{x}}_{p_{norm}}^T{\boldsymbol{x}}_{p_{norm}}$, which considers the contribution of the new sample to the constitution of the distorted correlation matrix, with a natural weight of $\frac{1}{n_g}$.
3. ${\overset{\sim }{\Sigma}}_g^{-1}{\Delta b}_g{\Delta b}_g^T{\overset{\sim }{\Sigma}}_g^{-1}$, which considers the effects of the distortion of Σ_*g*_ and *b*_*g*_ in ${\overset{\sim }{S}}_g$.

Finally, the distortion of the correlation matrix will be measured with the estimation of the deviation between *S*_*g*_ and ${\overset{\sim }{S}}_g$, using the metric $\varphi \left({S}_g,{\overset{\sim }{S}}_g\right)$ defined in Eq. (14). As previously mentioned, if the number of samples for the group *g* is large, the integration of *x*_*p*_ will barely cause a distortion in the correlation structure, even if it has different features compared to the samples in *X*_*g*_. For example, if *X*_*g*_ were composed of 200 samples, the natural relative weight of the mean vector (*b*_*c*_) for the construction of the distorted mean vector would be ~ 0.995, while the natural weight of the sample would (only) be ~ 0.005.

On the other hand, if the weights were calculated assuming that *X*_*g*_ is composed of few samples, that is, replacing *n*_*g*_ for ${n}_g^{red}$ (so that ${n}_g^{red}<{n}_g$) in the quotients to calculate the relative weights, these weights would be more even and provide a weighting factor for the calculation of the distorted correlation matrix using all the information contained in the original samples of *X*_*g*_ (in *b*_*g*_, Σ_*g*_, and *S*_*g*_). This is equivalent to finding a generatrix base of a few samples/patients (${n}_g^{red}$) that can represent all the characteristics of *X*_*g*_, incorporate ***x***_*p*_, and then evaluate the distortion caused to the correlation structure, providing an artificial dimensional reduction. For example, if the relative weights were calculated assuming that *X*_*g*_ is composed only of three samples that exhibit all the attributes of the original dataset (i.e., ${n}_g^{red}=3$), these weights would have the values of 0.75 and 0.25, respectively, for the calculation of the distorted mean vector.

The lower threshold for this artificial dimensional reduction could be found making ${n}_g^{red}=2$ in the calculation of the relative weights. If ${n}_g^{red}=1$, this would lead to leaving out all the information contained in *S*_*g*_ to the estimation of ${\overset{\sim }{S}}_g$ (see Eq. (30)). A similar result is obtained for the standard deviation (see Eq. (28)).

### Proposed classification rule

Now that the artificial dimensional reduction approach has been proposed, it will be used alongside the metric *φ* for the creation of a tool to classify new samples/patients into either the control or case group. The classifier will work under the assumption that a sample’s likelihood of belonging to either group is inversely proportional to the distortion caused by its incorporation into that group. This classification approach includes the following steps:

1. Store the new sample in ***x***_*p*_.
2. Define the “maximum artificial dimension” to be evaluated as $n\le \mathit{\min}\left({n}_c,{n}_v\right)\ \left(n\in {\mathbbm{z}}^{+}\right).$ Choose a dimension “step of change”, $\Delta  n\in {\mathbbm{z}}^{+}$, such as *n* − 2 is divisible by ∆*n*. Thus, $\frac{\left(n-2\right)}{\Delta  n}+1$ would define the number of artificial dimensions to be evaluated. Therefore, we set ${n}_g^{red}=\left(2,2+\Delta  n,2+2\Delta  n,\dots, n\right)$ for both *g* = *c* and *g* = *v*.
3. Evaluate Eqs. (18), (25), (26) and (30) using ${n}_g^{red}$ instead of *n*_*g*_. Perform this evaluation for both *g* = *c* and *g* = *v*, and for all values of ${n}_g^{red}$. Store the resulting distorted correlation matrices as

31 $$ {\overset{\sim }{\mathcal{S}}}_c=\left\{\begin{array}{c}{\overset{\sim }{S}}_{c_{\mid {}_{n_g^{red}=2}}}\\ {}\vdots \\ {}{\overset{\sim }{S}}_{c_{\mid_{n_g^{red}=n}}}\end{array}\right\},\kern1em {\overset{\sim }{\mathcal{S}}}_v=\left\{\begin{array}{c}{\overset{\sim }{S}}_{v_{\mid_{n_g^{red}=2}}}\\ {}\vdots \\ {}{\overset{\sim }{S}}_{v_{\mid_{n_g^{red}=n}}}\end{array}\right\} $$

4. For each ${n}_g^{red}=\left(2,2+\Delta  n,2+2\Delta  n,\dots, n\right)$, calculate
  32 $$ {\left.\left({\psi}_g\right)\right|}_{n_g^{red}}:= \frac{1}{\left|\varphi \left({S}_g,{\overset{\sim }{S}}_{g_{n_g^{red}}}\right)\right|},\kern1.75em g=\left\{c,v\right\} $$
  where |*l*| is the absolute value of *l*. In consequence, large values of *ψ* indicate a small distortion in the correlation structure, and therefore, a high degree of affinity between *X*_*g*_ and ***x***_*p*_. On the other hand, small values of *ψ* indicate a big distortion and a low degree of affinity between *X*_*g*_ and ***x***_*p*_.
5. Calculate the average value for ${\left.\left({\psi}_g\right)\right|}_{n_g^{red}}$ as

33 $$ {\overline{\psi}}_g=\frac{1}{n}\sum \limits_{\forall {n}_g^{red}}\left[{\left.\left({\psi}_g\right)\right|}_{n_g^{red}}\right],\kern1.75em g=\left\{c,v\right\} $$

**Fig. 4:**
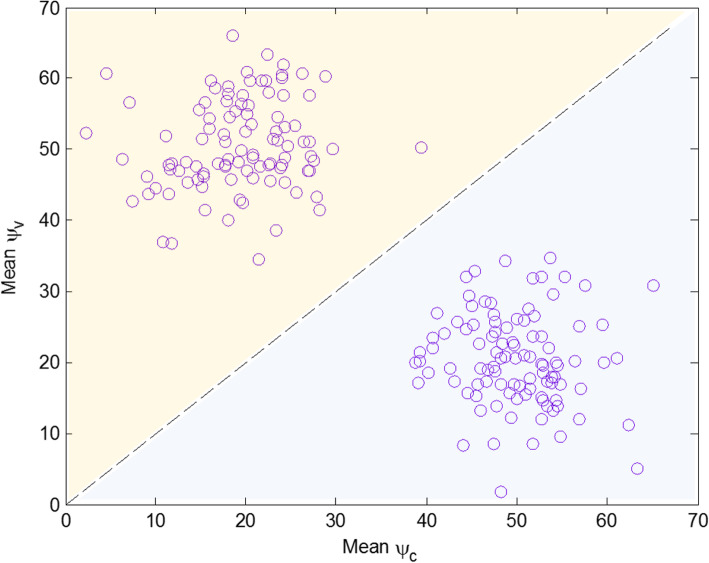
Illustration of new samples and the line that separates both groups with the proposed method. Samples lying in the upper semi-plane will be classified in the case (*v*) group and in the control (*c*) group otherwise

6. Finally, the outcomes of the proposed classification rule, for a single sample, are ${\overline{\psi}}_c$ and ${\overline{\psi}}_v$. The method will classify the sample into the group with the greater value of ${\overline{\psi}}_g$. Figure 4 shows a graphical representation to visualize the outcome of the proposed classification method after classifying a set of new samples one-by-one.

## Performance assessment with synthetic data

In this section, we assess the performance of the proposed method to correctly classify synthetically generated data.

### Synthetic data generation

We conducted *in silico* experiments to assess the performance of the proposed method under different parameter settings. The following procedure was used to generate synthetic datasets:

1. Define the quadruplet (*n*_*i*_, *m*_*j*_, *ρ*_*c*_, *ρ*_*v*_). Set *n* = {20,40,60,80,100,120,140,160}, *m* = {20,40,60,80,100,120,140}, *ρ*_*c*_ = 0.1, *ρ*_*v*_ = 0.2.
2. For every quadruplet in step 1 construct a pair of generatrix correlation matrices, ${\Sigma}_{c_{j,c}}$ and ${\Sigma}_{v_{j,v}}$ as ${\Sigma}_{c_{j,c}}=\left(1-{\rho}_c\right){I}_{m_j}+{\rho}_c{1}_{m_j}{1}_{m_j}^T$ and ${\Sigma}_{v_{j,v}}=\left(1-{\rho}_v\right){I}_{m_j}+{\rho}_v{1}_{m_j}{1}_{m_j}^T$, where ${I}_{m_j}\in {\mathbb{R}}^{m_j\times {m}_j}$ is the identity matrix and ${1}_{m_j}\in {\mathbb{R}}^{m_j\times 1}$ is column vector of ones.
3. For every pair $\left({\Sigma}_{c_{j,c}},{\Sigma}_{v_{j,v}}\right)$, *B* pairs of Normal-distributed matrices ${X}_{c_r}$ and ${X}_{v_r}$ (with *r* = {1, 2, …, *B*}) of dimension *n*_*i*_ × *m*_*j*_ are generated. For this purpose, the NumPy [54] Python package was used. The number of experimental replicates was *B* = 100.

### Performance assessment procedure

We used the correct classification rate (accuracy) as the assessment criterion to measure the performance of our method as follows:

1. Merge each $\left({X}_{c_r},{X}_{v_r}\right)$ into a single matrix $$ {X}_{Total}=\left[\begin{array}{c}{X}_{c_r}\\ {}{X}_{v_r}\end{array}\right]\in {\mathbb{R}}^{2n\times m} $$.
2. For every pair $\left({X}_{c_r},{X}_{v_r}\right)$, execute the proposed algorithm with each row sample ${x}_{p_i}={X}_{Tota{l}_i}\left[i,:\right]$, *i* = {1, 2, …, 2*n*}, and classify ${x}_{p_i}$.
3. Compute the average classification accuracy as:
  34 $$ \mathrm{Accuracy}=100\times \frac{N}{2n} $$
  where *N* is the number of correctly classified samples.

### Performance assessment results with synthetic data

Table 3 summarizes the main results. Our method exhibits exceptional accuracy for all the configurations tested. Interestingly, accuracy decreases as the number of features *m* decreases and the sample size *n* increases.

**Table 3:** Performance of the proposed method for synthetic datasets. Configurations (*n*, *m*) not reported showed 100% Classification Accuracy

Sample size (*n*)	Number of features (*m*)	Classification Accuracy (%)
80	40	99.8
100	20	98.1
120	20	99.7
160	20	98.0

## Validation with real datasets

In this section, we study the performance of the proposed method using two real-world datasets, which contain OTU count tables obtained through 16S rRNA gene sequencing data from microbiota experiments. We also compare the classification accuracy of our method with those of two state-of-the-art methods: SVM [39] and SVM-RFE [41].

### Datasets

The first dataset is from the American Gut Project (AGP) [51], which is one of the largest crowd-funded microbiome research projects. The second dataset is the Greengenes (GG) database [52], created with the PhyloChip 16s rRNA microarray. For the comparison experiment, only fractions of the datasets were used. In particular, a total of 578 samples and 127 features comprised the AGP data set, while 500 samples and 26 features comprised the GG data set. In both data sets, 50% of the samples correspond to cases.

### Validation scenarios results

Datasets were preprocessed as described in section “Data pretreatment”. Further, the proposed method, as well as the SVM and SVM-RFE methods, were applied after separating the whole data set into training, testing, and validation sets using 70, 20, and 10% of the data, respectively. For the SVM-RFE method, the number of features to select was ${n}_{features}=\left\{5,10,15,\frac{n_{features}}{2}\right\}$ and the average of the results was calculated. The tuning parameters used for the SVM and SVM-RFE methods were *C* = 1 and *γ* = 0.05, where *C* trades off the correct classification of training examples against the maximization of the decision function’s margin, and *γ* defines how far the influence of a single training example reaches.

Table 4 shows the main results. For the AGP data set, SVM is the least accurate, and SVM-RFE has the highest accuracy. This latter result is mostly due to all the strong features of SVM and the ability of the SVM-RFE method to eliminate variables that are not highly relevant in the data. Interestingly, our method outperforms SVM and is a close competitor of SVM-RFE.

**Table 4:** Classification accuracy for each method for the AGP and GG data sets

Dataset	SVM	SVM-RFE	Proposed Method
*AGP*	92.03%	96.33%	95.06%
*GG*	89.34%	92%	94%

**Fig. 5:**
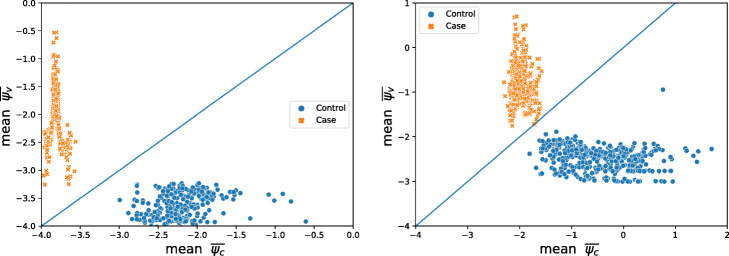
Illustration of new samples and the line that separates both groups with the proposed method for the AGP (left) and GG (right) data sets

For the GG dataset, although the number of variables is small, the SVM-RFE and our method showed accuracy values above 90%, while the accuracy for the SVM method is below this threshold. It is worth highlighting that, for this data set, our method outperforms both the SVM and SVM-RFE methods. The latter result is thanks to the artificial dimensional reduction conducted to balance the natural weights when the number of samples is greater than the number of variables. Figure 5 provides a graphical illustration of the proposed method’s classification outcome for both real datasets used for validation, i.e., the AGP and the GG.

## Discussion and conclusions

The ability to characterize populations of patients, species, or biological features, usually comprising a large number of variables in order to use the extracted characteristics to classify new samples into one of such populations’ categories is a relevant tool for biological and medical studies. When data describing these populations is compositional, further limitations and challenges arise.

Here, we proposed a new method to classify samples into one of two previously known categories. The method uses a new metric developed to quantify the overall correlation structure deviation between two datasets, and a new dimensionality reduction technique. Although we illustrated the usefulness of our proposal with compositional data, its application is not limited, under any circumstances, to data of this nature. In fact, when data is not compositional, the centered log-ratio transformation and the zero-replacement algorithm must not be applied.

Validation with synthetic data showed that the proposed method achieves accuracy values above 98%. Moreover, comparison of the performance of our method with that of SVM and the SVM-RFE (i.e., two state-of-the-art classification techniques), using two real-world datasets from 16 s RNA sequencing experiments, showed that our method outperforms the SVM method in both data sets, outperforms the SVM-RFE method in the GG data set, and is a close competitor of the SVM-RFE method in the AGP data set.

Future studies may address the ability of our proposed method to perform accurately for a broader range of dimensions (number of variables and samples) and assess its performance for more scenarios of dissimilar correlation structures other than that for *ρ*_*c*_ = 0.1 and *ρ*_*v*_ = 0.2. Moreover, our method may be extrapolated for multi-category classification, and a performance assessment may be conducted to test its classification accuracy in non-binary scenarios.

## Data Availability

The source code, implemented in Python 3, is readily available in the following GitHub site: https://github.com/JoaoRacedo/arn_seq_pipeline. This code generates synthetic datasets to demonstrate the use of the pipeline. The American Gut Project’s datasets can be found on the following website: http://americangut.org. Finally, the Greengenes’ datasets can be found on: https://greengenes.lbl.gov/Download/OTUs/.

## Abbreviations

AGP: American gut project
alr: Additive lo-ratio
ANN: Artificial Neural Networks
BM: Bayesian multiplicative (algorithm)
clr: Centered log-ratio
CSVM-RMFE: Correlation based support vector machine–recursive multiple feature elimination
GG: Greengenes (database)
GrBoost: Gradient boosting
ilr: Isometric log-ratio
NN: Nearest Neighbor
OTU: Operational taxonomic unit
PCA: Principal component analysis
rRNA: Ribosomal ribonucleic acid
SPIEC-EASI: Sparse inverse covariance estimation for ecological association inference
SVM: Support vector machine
SVM-RFE: Support vector machine recursive feature elimination
